# Effects of COVID-19 Vaccination Timing and Risk Prioritization on Mortality Rates, United States

**DOI:** 10.3201/eid2707.210118

**Published:** 2021-07

**Authors:** Xutong Wang, Zhanwei Du, Kaitlyn E. Johnson, Remy F. Pasco, Spencer J. Fox, Michael Lachmann, Jason S. McLellan, Lauren Ancel Meyers

**Affiliations:** The University of Texas at Austin, Austin, Texas, USA (X. Wang, Z. Du, K.E. Johnson, R.F. Pasco, S.J. Fox, J.S. McLellan, L.A. Meyers);; The University of Hong Kong, Hong Kong, China (Z. Du);; Hong Kong Science and Technology Park, Hong Kong (Z. Du);; Santa Fe Institute, Santa Fe, New Mexico, USA (M. Lachmann, L.A. Meyers)

**Keywords:** COVID-19, SARS-CoV-2, severe acute respiratory syndrome coronavirus 2, viruses, respiratory infections, zoonoses, vaccines, mortality, pandemics, epidemiology, mathematical model, United States, Texas, coronavirus disease

## Abstract

During rollout of coronavirus disease vaccination, policymakers have faced critical trade-offs. Using a mathematical model of transmission, we found that timing of vaccination rollout would be expected to have a substantially greater effect on mortality rate than risk-based prioritization and uptake and that prioritizing first doses over second doses may be lifesaving.

In December 2020, the US government issued emergency use authorization for two 2-dose severe acute respiratory syndrome coronavirus 2 (SARS-CoV-2) vaccines, both estimated to be >94% efficacious in preventing symptomatic coronavirus disease (COVID-19) (*1*–*3*). The Advisory Committee on Immunization Practices immediately recommended the prioritization of frontline workers and high-risk subgroups (*4*). As of February 14, 2021, ≈52 million doses have been administered (*5*). We used a mathematical model of COVID-19 transmission to evaluate the effects of vaccine timing, risk prioritization, number of doses administered, and uptake rates on population-level mortality rates (Figure).

**Figure:**
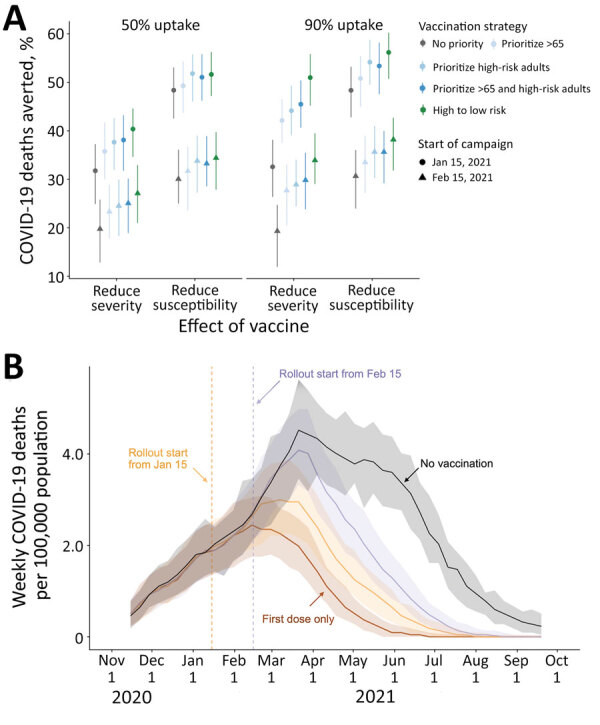
Projected COVID-19 deaths and deaths averted in the Austin–Round Rock Metropolitan Statistical Area (Austin, TX, USA) under various vaccine rollout scenarios for November 8, 2020–September 17, 2021. A) COVID-19 deaths averted after January 15, 2021, under combinations of vaccine uptake of 50% (left) or 90% (right); type of protection, either infection blocking (reducing susceptibility) or symptom blocking (reducing severity); rollout dates, either January 15 (circles) or February 15 (triangles); and risk prioritization, either no priority (gray), prioritize all adults >65 years of age (light blue), adults with high-risk underlying conditions (medium blue), or the combination of the two (dark blue), or a 10-phase risk-ordered strategy (green) that sequentially vaccinates >65 y high risk, 50–64 y high risk, >65 y low risk, 18–49 y high risk, 50–64 y low risk, 18–49 y low risk, 0–4 y high risk, 5–17 y high risk, 0–4 y low risk, 5–17 y low risk. Points and whiskers indicate the median and 95% CI across 200 paired stochastic simulations. B) Weekly incident COVID-19 deaths per 100,000 population, assuming intermediate (70%) uptake (*6*) without vaccine (black) or under a 10-phase risk-based rollout of a 95% efficacious infection-blocking vaccine, starting either January 15 (orange) or February 15 (purple). The brown line assumes that only first doses are administered starting January 15. Solid lines and shading indicate the median and 95% CI across 200 stochastic simulations. COVID-19, coronavirus disease.

Focusing on Austin, Texas, USA, we projected COVID-19 deaths over 8 months for both an infection-blocking vaccine that prevents infection upon exposure (assuming 95% reduction in susceptibility in vaccinated persons) and a symptom-blocking vaccine that prevents symptoms upon infection (assuming 95% reduction in symptomatic ratio in vaccinated persons). Vaccination would begin on January 15 or February 15, with 10,000 vaccines administered weekly and allocated to cities pro rata. We compare 3 strategies: no priority groups; 1 of 3 priority groups vaccinated before the general public (adults >65 years of age, adults who have high-risk underlying conditions, or both); and 10 phases that vaccinate age–risk groups in order of risk for severe COVID-19 outcomes. Stochastic simulations assumed that 7.6% of the overall population of the Austin–Round Rock Metropolitan Statistical Area were immunized by infection before January 15.

If a perfectly risk-prioritized (10-phase) rollout of an infection-blocking vaccine were to begin January 15, we estimated that 52% (95% CI 47%–56%) of deaths would be averted relative to the baseline of no vaccines, assuming 50% uptake, or 56% (95% CI 51%–60%) of deaths averted assuming 90% uptake (Figure, panel A). If rollout were delayed 1 month, 34% (95% CI 28%–40%) of deaths would be averted at 50% uptake, or 38% (95% CI 32%–43%) at 90% uptake. Under low (50%) uptake, prioritization has minimal benefit. Under high uptake (90%), the 10-stage strategy is optimal, followed by prioritizing adults >65 years of age and high-risk younger adults. 

Expected differences are magnified with a symptom-blocking vaccine. For a January 15 start and 50% uptake, the risk-prioritized 10-phase strategy would avert 40% (95% CI 35%–45%) of deaths, whereas unprioritized rollout would avert 32% (95% CI 25%–37%). If a single dose with 82% efficacy (*1*,*2*) is administered under the 10-phase strategy, we would expect a 50% (95% CI 45%–54%) reduction in mortality for a symptom-blocking vaccine and 66% (95% CI 63%–70%) reduction for an infection-blocking vaccine (Appendix Table 1).

These projections validate prioritizing high-risk groups. In a pessimistic scenario in which a symptom-blocking vaccine rollout began in February 2021 with 50% uptake, prioritizing high-risk adults and adults >65 would avert ≈17,000 (95% CI 0–36,000) more deaths in the United States than a nonprioritized campaign. Given the state of the pandemic in early 2021, we expected vaccine delays to cost more lives than either imperfect prioritization or vaccine hesitancy. 

The United Kingdom and Belgium have prioritized first doses over second doses (*7*), in an effort to provide partial immunity to more persons. The United States has publicly resisted this approach, citing the lack of clinical trial data validating the approach (*8*). We found that providing a single (82% efficacious) dose would be expected to save more lives than the corresponding 2-dose strategy, because partially immunizing a large number confers a greater degree of population-level protection than more fully immunizing half as many. Although a 1-dose campaign may accelerate herd immunity and require far fewer resources than a 2-dose campaign, we strongly caution that additional data and single-dose trials are needed to establish efficacy. If the single-dose efficacy is <82%, then we would expect the difference between a single-dose strategy and the corresponding 2-dose strategy to be smaller. We expect similar reductions in mortality rate from both strategies when the single-dose efficacy is 52% (*2*) (Appendix Figure 7). We note that low-efficacy vaccines may increase the risk for vaccine-resistant variants (*9*) and that there may be political, commercial, and societal barriers to shifting priorities mid-campaign (*10*).

We assumed that vaccines provide lasting immunity and block either infection or symptoms, whereas the reality may be a hybrid of both (Appendix Table 2, Figure 2), along with riskier behavior stemming from pandemic weariness or overconfidence in the vaccination campaign. Our estimates reflect conditions in the United States in early 2021, as cases were surging toward a pandemic peak in the absence of effective mitigation. The estimated public health benefits of vaccines decrease under higher COVID-19 transmission rates that might occur with relaxed mitigation measures, lower levels of immunity before the rollout, or the emergence of more transmissible SARS-CoV-2 variants including B.1.1.7 (Appendix).

Risk prioritization is a valid approach for maximizing the impact of vaccines, but not at the expense of vaccination speed. Our projections suggest 2 immediate strategies: hybrid distributions that combine active outreach to priority groups with passive distribution to the general public; and distribution of single doses to as much of the population as possible, foregoing plans to hold second doses in reserve.

Appendix Additional information about modeled effects of coronavirus vaccination and prioritization on mortality, United States.
