# Supplementary material for: Effects of COVID-19 Vaccination Timing and Risk Prioritization on Mortality Rates, United States
Source: Emerg Infect Dis. 2021 Jul;27(7):1976–9. doi: 10.3201/eid2707.210118 (PMC8237869; doi:10.3201/eid2707.210118)
Supplement: Appendix — Additional information about modeled effects of coronavirus vaccination and prioritization on mortality, United States. [file 21-0118-Techapp-s1.pdf]

Article DOI: https://doi.org/10.3201/eid2707.210118

Effects of COVID-19 Vaccination Timing and Risk Prioritization on Mortality Rates, United States

Appendix

Hybrid Scenarios: Infection-Blocking and Symptom-Blocking Vaccine

Our model assumes the vaccine is either infection blocking or symptom blocking, while the reality may be somewhere in between. Therefore, we projected coronavirus disease (COVID-19) mortality under a hybrid scenario where 2 doses of the vaccine are 67% efficacious against all infections and 82% efficacious against developing symptoms (Appendix Table 2, Appendix Table 6, Appendix Figure 2).

Stochastic Compartmental Model of COVID-19 Transmission in the Austin-Round Rock Metropolitan Area

Appendix Figure 3 includes a diagram of the model structure. For each vaccine, age and risk group, we build a separate set of compartments to model the transitions between the states: susceptible (S), exposed (E), pre-symptomatic infectious (I^P), symptomatic infectious (I^Y), asymptomatic infectious (I^A), symptomatic infectious that are hospitalized (I^H), recovered (R), and deceased (D). The symbols S, E, I^P, I^Y, I^A, I^H, R, and D denote the number of persons in that state in the given vaccine/age/risk group and the total size of the vaccine/age/risk group is $N = S + E + I^P + I^Y + I^A + I^H + R + D$.

The model for individuals in vaccine group v, age group a, and risk group r is given by:

$$\frac{dS_{v,a,r}}{dt} = -\sum_{u \in V} \sum_{i \in A} \sum_{j \in K} S_{v,a,r} \kappa_{v,a,r} \beta_0 \phi_{a,i} \left(\omega_u^P I_{u,i,j}^P + \omega_u^A I_{u,i,j}^A + \omega_u^Y I_{u,i,j}^Y \right) / N_i$$

$$\frac{dE_{v,a,r}}{dt} = \sum_{u \in V} \sum_{i \in A} \sum_{j \in K} S_{v,a,r} \kappa_{v,a,r} \beta_0 \phi_{a,i} \left(\omega_u^P I_{u,i,j}^P + \omega_u^A I_{u,i,j}^A + \omega_u^Y I_{u,i,j}^Y \right) / N_i - \sigma E_{v,a,r}$$

$$\frac{dI^A_{v,a,r}}{dt} = \left(1 - \lambda_{v,a,r}\tau\right)\sigma E_{v,a,r} - \gamma^A I^A_{v,a,r}$$

$$\frac{dI^P_{v,a,r}}{dt} = \lambda_{v,a,r}\tau\sigma E_{v,a,r} - \rho I^P_{v,a,r}$$

$$\frac{dI^Y_{v,a,r}}{dt} = \rho I^P_{v,a,r} - (1-\pi)\gamma^Y I^Y_{v,a,r} - \pi\eta I^Y_{v,a,r}$$

$$\frac{dI^H_{v,a,r}}{dt} = \pi\eta I^Y_{v,a,r} - (1-\nu)\gamma^H I^H_{v,a,r} - \nu\mu I^H_{v,a,r}$$

$$\frac{dR_{v,a,r}}{dt} = \gamma^A I^A_{v,a,r} + (1-\pi)\gamma^Y I^Y_{v,a,r} + (1-\nu)\gamma^H I^H_{v,a,r}$$

$$\frac{dD_{v,a,r}}{dt} = \nu\mu I^H_{v,a,r}$$

where V, A and K are all possible vaccine, age and risk groups, $\omega^A,\ \omega^Y,\ \omega^P$ are relative infectiousness of the $I^A,\ I^Y,\ I^P$ compartments, respectively, β_0 is baseline transmission rate, $\phi_{a,i}$ is the mixing rate between age group $a,\ i\ \in\ A$, $\gamma^A, \gamma^Y,\ \gamma^H$ are the recovery rates for the $I^A,\ I^Y,\ I^H$ compartments, respectively, σ is the exposed rate, τ is the symptomatic ratio, ρ is the rate from pre-symptomatic to symptomatic, π is the proportion of symptomatic individuals requiring hospitalization, η is rate at which hospitalized cases enter the hospital following symptom onset, ν is mortality rate for hospitalized cases, and μ is rate at which terminal patients die.

The transition between each vaccine group v, is given by

$$\frac{dX_{U,a,r}}{dt} = -\alpha^i_{a,r} X_{U,a,r}$$

$$\frac{dX_{W^i,a,r}}{dt} = \alpha^i_{a,r} X_{U,a,r} - \delta^i X_{W^i,a,r}$$

$$\frac{dX_{V^i,a,r}}{dt} = \delta^i X_{W^i,a,r} - \alpha^{ii}_{a,r}\delta X_{V^i,a,r}$$

$$\frac{dX_{W^{ii},a,r}}{dt} = \alpha^{ii}_{a,r}\delta X_{V^i,a,r} - \delta^{ii} X_{W^{ii},a,r}$$

$$\frac{dX_{V^{ii},a,r}}{dt} = \delta^{ii} X_{W^{ii},a,r}$$

where $X \in \{S, E, I^A, I^P, I^Y, I^H, R, D\}$, and $v \in \{U, W^i, V^i, W^{ii}, V^{ii}\}$, α^i is vaccination rate, δ^i is the rate that individuals that receive the first injection gain partial immunity, α^{ii} is second injection adherence rate, δ is the delay in second dose injection, δ^{ii} is the rate that individuals that receive the second injection gain immunity.

We model stochastic transitions between compartments using the τ-leap method (*3*,*4*) with key parameters given in Appendix Tables 4–11. Assuming that the events at each time-step are independent and do not impact the underlying transition rates, the numbers of each type of event should follow Poisson distributions with means equal to the rate parameters. We thus simulate the model according to the following equations:

$$S_{v,a,r}(t+1) - S_{v,a,r}(t) = -P_1$$

$$E_{v,a,r}(t+1) - E_{v,a,r}(t) = P_1 - P_2$$

$$I^A_{v,a,r}(t+1) - I^A_{v,a,r}(t) = \left(1 - \lambda_{v,a,r}\tau\right)P_2 - P_3$$

$$I^P_{v,a,r}(t+1) - I^P_{v,a,r}(t) = \lambda_{v,a,r}\tau P_2 - P_4$$

$$I^Y_{v,a,r}(t+1) - I^Y_{v,a,r}(t) = P_4 - P_5 - P_6$$

$$I^H_{v,a,r}(t+1) - I^H_{v,a,r}(t) = P_6 - P_7 - P_8$$

$$D_{v,a,r}(t+1) - D_{v,a,r}(t) = P_7$$

$$R_{v,a,r}(t+1) - R_{v,a,r}(t) = P_3 + P_5 + P_8$$

$$X_{U,a,r}(t+1) - X_{U,a,r}(t) = -\alpha^i_{a,r}(t)X_{U,a,r}(t)$$

$$X_{W^i,a,r}(t+1) - X_{W^i,a,r}(t) = \alpha^i_{a,r}(t)X_{U,a,r}(t) - P_9$$

$$X_{V^i,a,r}(t+1) - X_{V^i,a,r}(t) = P_9 - P_{10}$$

$$X_{W^{ii},a,r}(t+1) - X_{W^{ii},a,r}(t) = P_{10} - P_{11}$$

$$X_{V^{ii},a,r}(t+1) - X_{V^{ii},a,r}(t) = P_{11}$$

with

$$P_1 \sim Pois\left(S_{v,a,r}(t)F_{v,a,r}(t)\right)$$

$$P_2 \sim Pois\left(\sigma E_{v,a,r}(t)\right)$$

$$P_3 \sim Pois\left(\gamma^A I^A_{v,a,r}(t)\right)$$

$$P_4 \sim Pois\left(\rho I^P_{v,a,r}(t)\right)$$

$$P_5 \sim Pois\left((1-\pi)\gamma^Y I^Y_{v,a,r}(t)\right)$$

$$P_6 \sim Pois\left(\pi\eta I^Y_{v,a,r}(t)\right)$$

$$P_7 \sim Pois\left(\nu\mu I^H_{v,a,r}(t)\right)$$

$$P_8 \sim Pois\left((1-\nu)\gamma^H I^H_{v,a,r}(t)\right)$$

$$P_9 \sim Pois\left(\delta^i X_{W^i,a,r}(t)\right)$$

$$P_{10} \sim Pois\left(\alpha^{ii}\delta X_{V^i,a,r}(t)\right)$$

$$P_{11} \sim Pois\left(\delta^{ii} X_{W^{ii},a,r}(t)\right)$$

and where $F_{v,a,r}$ denotes the force of infection for individuals in vaccine group *v*, age group *a*, and risk group *r* and is given by:

$$F_{v,a,r}(t) = \sum_{u\in V}\sum_{i\in A}\sum_{j\in K}\kappa_{v,a,r}\beta_0\phi_{a,i}\left(\omega^P_u I^P_{u,i,j} + \omega^A_u I^A_{u,i,j} + \omega^Y_u I^Y_{u,i,j}\right)/N_i.$$

Model Parameters

We developed a compartmental model of COVID-19 transmission in a US city, the Austin–Round Rock Metropolitan Statistical Area, using a model period of November 8, 2020−September 17, 2021. We applied various vaccine rollout scenarios and modeled each age-risk subgroup with a separate set of compartments. (Appendix Figure 3). Upon infection, susceptible persons progress to exposed and then to either presymptomatic infectious or asymptomatic infectious. All asymptomatic cases eventually progress to a recovered class where they remain protected from future infection; presymptomatic cases progress to symptomatic then either are hospitalized or recover. Mortality varies by age group and risk group and is assumed to be preceded by hospitalization. Within each compartment, individuals are divided by vaccination status: unvaccinated, newly vaccinated with the first dose, vaccinated with the first dose, newly

vaccinated with the second dose, and fully vaccinated with the second dose. We modeled stochastic transitions between compartments and sample vaccine efficacy parameters from distributions to capture uncertainty, while keeping all other parameters fixed.

Initial Conditions

Initial conditions were derived using a COVID-19 healthcare forecasting model that we developed in a partnership with the city of Austin and use to provide daily transmission and healthcare projections on a public dashboard (*5*). The forecasting model is almost identical to the model in this study, but without vaccines. Specifically, it is an expanded stochastic SEIR model with 8 disease progression compartments, including symptomatic, presymptomatic, asymptomatic patients, and hospitalization. The population is divided into 5 age groups, with different rates of contacts within and between age groups, a high-risk category with each age group, and age- and risk-specific rates of hospitalization. The demographic, health, and mixing parameters are identical to those assumed in this study.

To make daily dashboard projections, we incorporate anonymized local mobility data from SafeGraph (S. Gao et al., unpub. data, http://arxiv.org/abs/2004.04544) into transmission rate. We assume published estimates for all disease progression parameters and calibrate the remaining unknown states and parameters to local COVID-19 hospital admissions and discharge data using iterated filtering made available through the POMP R package (*39*). The result of the statistical inference is posterior densities for parameters governing the impact of mobility on transmission and the reporting process of hospitalization data, and for hidden states of the model including the number of infected persons (*40*).

To obtain initial conditions for this study, we used the states in the fitted model based on data through November 7, 2020 (Appendix Table 11).

Sensitivity Analyses

Sensitivity Analysis with Respect to the Effective Reproduction Number (R_e) of the Virus on November 8, 2021

Our base scenarios assume an effective reproductive number (R_e) of 1.2 (*5*). Here, we provide projections based on 3 alternative transmission rate scenarios: R_e = 1.5, R_e = 1.05, and R_e

increasing linearly from 1.2 to 1.8 by May 1, 2021, and remaining at 1.8 through September 17, 2021.

Under the lowest transmission rate scenario (R_e = 1.05), the pace of the epidemic is slower and the expected effect of vaccinations on overall mortality and the duration of the pandemic is greater than in the base case (Appendix Figure 4). Under the moderate transmission scenario (R_e = 1.5), the reverse occurs. The pandemic sweeps through and achieves herd immunity rapidly, leaving little opportunity for vaccines to prevent infections and deaths.

The increasing transmission rate scenario roughly models the emergence and rapid spread of a more transmissible SARS-CoV-2 variant, like B.1.1.7. The pandemic wave reaches a peak at a similar time to the base case, but reaches a much higher prevalence of COVID-19. Overall, vaccination has a lower impact on COVID-19 mortality and the duration of the pandemic wave, but risk-based prioritization and community uptake have similar relative impacts.

Consider the best of the vaccination scenarios: 90% uptake of a perfectly-prioritized rollout of a vaccine that reduces susceptibility beginning on January 15, 2021. The expected COVID-19 deaths averted between January 15 and September 17, 2021 are 60% (95% CI 51%–67%) for R_e = 1.05, 56% (95% CI 51%–60%) for R_e = 1.2, 22% (95% CI 17%–25%) for R_e = 1.5, and 41% for dynamic R_e scenarios. The expected numbers of COVID-19 deaths per 100,000 population during the peak week are 27 (95% CI 22–33) for R_e = 1.05, 107 (95% CI 100–122) for R_e = 1.2, 386 (95% CI 370–407) for R_e = 1.5; and 439 (95% CI 413–474) for dynamic R_e.

Sensitivity Analysis with Respect to Prior Immunity and the Prevalence of Infections as of November 8, 2020

Our base scenarios assume that 7.6% of the population have obtained immunity due to prior infection by early November and that 0.3% of the population is infected on the first day of the simulation (November 8, 2020) (Appendix Table 11) (*5*). In Figure 5, we provide projections assuming that twice as many persons are infected (0.6%) are infected at the start of each simulation or twice as many persons (15.2%) are immune at the outset. All projections assume the same transmission rate per contact, which was derived to produce a reproduction number of R_e = 1.2 in the absence of prior immunity (Appendix Table 5).

Sensitivity Analysis with Respect to Vaccine Efficacy

Our original analyses assume 95% vaccine efficacy following 2 doses and 82% efficacy following a single dose (Appendix Table 6). However, recent studies have raised concerns that some SARS-CoV-2 vaccines may have reduced efficacy against emerging and future variants, including B.1.351 (*41*). We provide projections assuming that the vaccine efficacy is reduced by 50% (Appendix Figure 6).

Sensitivity Analysis with Respect to Vaccine Efficacy after a Single Dose

Our baseline analyses assume 82% vaccine efficacy following a single dose (Figure panel C, Appendix Table 6). However, a recent commentary (*42*) suggests that the efficacy following a single dose of the Pfizer-BioNTech vaccine may be even higher than 82% and provides revised estimates that exclude trial data (*35*) from the 2 weeks immediately following the first injection, while the body is still building an immune response. Appendix Figure 7 provides projections assuming single-dose efficacies of 52.4% (95% CI 29.5%–68.4%), 68.5% (95% CI 46.5%–81.5%), 82% (95% CI 75.6%–86.9%), and 92.6% (95% CI 69%–98.3%) (*42*).

Estimation of Age-Stratified Proportion of Population at High Risk for COVID-19 Complications

We estimate age-specific proportions of the population at high risk for complications from COVID-19 based on data for Austin, Texas and Round Rock, Texas from the CDC's 500 Cities Project (Appendix Figure 8) (*16*). We assume that high risk conditions for COVID-19 are the same as those specified for influenza by the CDC (*13*). CDC's 500 Cities Project provides city-specific estimates of prevalence for several of these conditions among adults (*17*). The estimates were obtained from the 2015–2016 Behavioral Risk Factor Surveillance System (BRFSS) data using a small-area estimation methodology called multilevel regression and poststratification (*14,15*). It links geocoded health surveys to high spatial resolution population demographic and socioeconomic data (*14*).

Estimating High-Risk Proportions for Adults

To estimate the proportion of adults at high risk for complications, we use the CDC's 500 Cities data, as well as data on the prevalence of HIV/AIDS, obesity, and pregnancy among adults (Appendix Table 12).

The CDC 500 cities dataset includes the prevalence of each condition on its own, rather than the prevalence of multiple conditions (e.g., dyads or triads). Thus, we use separate co-morbidity estimates to determine overlap. Reference about chronic conditions (*18*) gives US estimates for the proportion of the adult population with 0, 1, or $\geq$2 chronic conditions, per age group. Using this and the 500 cities data we can estimate the proportion of the population pHR in each age group in each city with $\geq$1 chronic condition listed in the CDC 500 cities data (Appendix Table 12) putting them at high risk for flu complications.

HIV

We use the data from Table 20a in CDC HIV Surveillance Report (*19*) to estimate the population in each risk group living with HIV in the United States (last column, 2015 data). Assuming independence between HIV and other chronic conditions, we increase the proportion of the population at high-risk for influenza to account for persons living with HIV but no other underlying conditions.

Morbid Obesity

A BMI >40 kg/m^2 indicates morbid obesity and is considered high risk for influenza. The 500 Cities Project reports the prevalence of obese persons in each city with BMI over 30 kg/m^2 (not necessarily morbid obesity). We used the data from Table 1 in Sturm and Hattori (*20*) to estimate the proportion of people with BMI>30 that actually have BMI>40 (across the United States); we then apply this to the 500 Cities obesity data to estimate the proportion of persons who are morbidly obese in each city. Table 1 of Morgan et al. (*21*) suggests that 51.2% of morbidly obese adults have $\geq$1 other high-risk chronic condition, and update our high-risk population estimates accordingly to account for overlap.

Pregnancy

We estimated the number of pregnant women in each age group and each city, following the methodology in CDC Reproductive Health Report (*22*). We assume independence between any of the high-risk factors and pregnancy, and further assume that half the population are women.

Estimating High-Risk Proportions for Children

Because the 500 Cities Project reports data for only adults >18 years of age, we took a different approach to estimating the proportion of children at high risk for severe influenza. The 2 most prevalent risk factors for children are asthma and obesity; we also accounted for childhood diabetes, HIV, and cancer.

From Miller et al. (*23*), we obtained national estimates of chronic conditions in children. For asthma, we assumed that variation among cities will be similar for children and adults. Thus, we used the relative prevalences of asthma in adults to scale our estimates for children in each city. The prevalence of HIV in children are taken from CDC HIV surveillance report (*19*) and the prevalence of cancer from the CDC cancer research report (*24*).

We first estimated the proportion of children who had asthma, diabetes, cancer, or HIV (assuming no overlap in these conditions). We estimated city-level morbid obesity in children using the estimated morbid obesity in adults multiplied by a national constant ratio for each age group estimated from Hales et al. (*25*); this ratio represents the prevalence in morbid obesity in children given the prevalence observed in adults. From Morgan et al. (*21*), we estimated that 25% of morbidly obese children have another high-risk condition and adjusted our final estimates accordingly.

Resulting Estimates

We compared our estimates for the Austin–Round Rock Metropolitan Area to published national-level estimates (*26*) of the proportion of each age group with underlying high-risk conditions (Appendix Table 13). The biggest difference was observed in older adults; Austin had a lower proportion at risk for complications for COVID-19 than the national average; for the 25–39 year age group, the high-risk proportion was slightly higher than the national average.

References

1. Texas Department of State Health Services. COVID-19 vaccine allocations. [cited 2021 Feb 4]. https://dshs.texas.gov/coronavirus/immunize/vaccineallocations.aspx

2. U.S. public now divided over whether to get COVID-19 vaccine. 2020 [cited 2020 Dec 14]. https://www.pewresearch.org/science/2020/09/17/u-s-public-now-divided-over-whether-to-get-covid-19-vaccine

3. Keeling MJ, Rohani P. Modeling infectious diseases in humans and animals. Princeton University Press; 2011. 408 p.

4. Gillespie DT. Approximate accelerated stochastic simulation of chemically reacting systems. J Chem Phys. 2001;115:1716–33. https://doi.org/10.1063/1.1378322

5. University of Texas COVID-19 Modeling Consortium. COVID-19 healthcare forecasts: Austin, Texas [cited 2020 Dec 8]. https://covid-19.tacc.utexas.edu/dashboards/austin

6. Austin Independent School District. Calendar of events. [cited 2020 Mar 26]. https://www.austinisd.org/calendar

7. Prem K, Cook AR, Jit M. Projecting social contact matrices in 152 countries using contact surveys and demographic data. PLoS Comput Biol. 2017;13:e1005697. https://doi.org/10.1371/journal.pcbi.1005697 PubMed

8. Mossong J, Hens N, Jit M, Beutels P, Auranen K, Mikolajczyk R, et al. Social contacts and mixing patterns relevant to the spread of infectious diseases. PLoS Med. 2008;5:e74. https://doi.org/10.1371/journal.pmed.0050074 PubMed

9. Diekmann O, Heesterbeek JAP, Roberts MG. The construction of next-generation matrices for compartmental epidemic models. J R Soc Interface. 2010;7:873–85. https://doi.org/10.1098/rsif.2009.0386 PubMed

10. He X, Lau EHY, Wu P, Deng X, Wang J, Hao X, et al. Temporal dynamics in viral shedding and transmissibility of COVID-19. Nat Med. 2020;26:672–5. PubMed https://doi.org/10.1038/s41591-020-0869-5

11. Gudbjartsson DF, Helgason A, Jonsson H, Magnusson OT, Melsted P, Norddahl GL, et al. Spread of SARS-CoV-2 in the Icelandic population. N Engl J Med. 2020;382:2302–15. PubMed https://doi.org/10.1056/NEJMoa2006100

12. Zhang J, Litvinova M, Wang W, Wang Y, Deng X, Chen X, et al. Evolving epidemiology and transmission dynamics of coronavirus disease 2019 outside Hubei province, China: a descriptive and modelling study. Lancet Infect Dis. 2020;20:793–802. PubMed https://doi.org/10.1016/S1473-3099(20)30230-9

13. US Centers for Disease Control and Prevention. People at high risk for flu complications. 2019 [cited 2020 Mar 26]. https://www.cdc.gov/flu/highrisk/index.htm

14. US Centers for Disease Control and Prevention. Behavioral Risk Factor Surveillance System. 2019 [cited 2020 Mar 26]. https://www.cdc.gov/brfss/index.html

15. Zhang X, Holt JB, Lu H, Wheaton AG, Ford ES, Greenlund KJ, et al. Multilevel regression and poststratification for small-area estimation of population health outcomes: a case study of chronic obstructive pulmonary disease prevalence using the behavioral risk factor surveillance system. Am J Epidemiol. 2014;179:1025–33. https://doi.org/10.1093/aje/kwu018 PubMed

16. US Centers for Disease Control and Prevention. 500 Cities Project: local data for better health. 2019 [cited 2020 Jun 22]. https://www.cdc.gov/places/about/500-cities-2016-2019/index.html

17. US Centers for Disease Control and Prevention. Health Outcomes | 500 Cities. 2019 [cited 2020 Jun 22]. https://www.cdc.gov/500cities/definitions/health-outcomes.htm

18. Fox S, Duggan M. Who lives with chronic conditions. 2013 [cited 2020 Jun 22]. https://www.pewresearch.org/internet/2013/11/26/part-one-who-lives-with-chronic-conditions/

19. US Centers for Disease Control and Prevention. HIV surveillance report. 2016; 28. http://www.cdc gov/hiv/library/reports/hiv-surveillance html

20. Sturm R, Hattori A. Morbid obesity rates continue to rise rapidly in the United States. Int J Obes (Lond). 2013;37:889–91. PubMed https://doi.org/10.1038/ijo.2012.159

21. Morgan OW, Bramley A, Fowlkes A, Freedman DS, Taylor TH, Gargiullo P, et al. Morbid obesity as a risk factor for hospitalization and death due to 2009 pandemic influenza A(H1N1) disease. PLoS One. 2010;5:e9694. PubMed https://doi.org/10.1371/journal.pone.0009694

22. US Centers for Disease Control and Prevention. Estimating the number of pregnant women in a geographic area. [cited 2021 May 10]. https://www.cdc.gov/reproductivehealth/emergency/pdfs/PregnacyEstimatoBrochure508.pdf

23. Miller GF, Coffield E, Leroy Z, Wallin R. Prevalence and costs of five chronic conditions in children. J Sch Nurs. 2016;32:357–64. PubMed https://doi.org/10.1177/1059840516641190

24. American Cancer Society. Cancer facts & figures 2014. 2014 [cited 2020 Jun 22]. https://www.cancer.org/research/cancer-facts-statistics/all-cancer-facts-figures/cancer-facts-figures-2014.html

25. Hales CM, Fryar CD, Carroll MD, Freedman DS, Ogden CL. Trends in obesity and severe obesity prevalence in US youth and adults by sex and age, 2007–2008 to 2015–2016. JAMA. 2018;319:1723–5. PubMed https://doi.org/10.1001/jama.2018.3060

26. Zimmerman RK, Lauderdale DS, Tan SM, Wagener DK. Prevalence of high-risk indications for influenza vaccine varies by age, race, and income. Vaccine. 2010;28:6470–7. PubMed https://doi.org/10.1016/j.vaccine.2010.07.037

27. Martin JA, Hamilton BE, Osterman MJK, Driscoll AK, Drake P. Births: final data for 2017. Nat Vital Stati Rep. 2018;67:1–50.

28. Jatlaoui TC, Boutot ME, Mandel MG, Whiteman MK, Ti A, Petersen E, et al. Abortion surveillance—United States, 2015. MMWR Surveill Summ. 2018;67:1–45. PubMed https://doi.org/10.15585/mmwr.ss6713a1

29. Ventura SJ, Curtin SC, Abma JC, Henshaw SK. Estimated pregnancy rates and rates of pregnancy outcomes for the United States, 1990–2008. Natl Vital Stat Rep. 2012;60:1–21. PubMed

30. US Centers for Disease Control and Prevention. COVID-19 Pandemic planning scenarios. 2020 [cited 2021 Feb 13]. https://www.cdc.gov/coronavirus/2019-ncov/hcp/planning-scenarios.html

31. US Centers for Disease Control and Prevention. Evidence used to update the list of underlying medical conditions that increase a person's risk of severe illness from COVID-19. 2020 [cited 2021 Jan 15]. https://www.cdc.gov/coronavirus/2019-ncov/need-extra-precautions/evidence-table.html

32. Richardson S, Hirsch JS, Narasimhan M, Crawford JM, McGinn T, Davidson KW, et al.; the Northwell COVID-19 Research Consortium. Presenting characteristics, comorbidities, and outcomes among 5700 patients hospitalized with COVID-19 in the New York City area. JAMA. 2020;323:2052–9. PubMed https://doi.org/10.1001/jama.2020.6775

33. Lewnard JA, Liu VX, Jackson ML, Schmidt MA, Jewell BL, Flores JP, et al. Incidence, clinical outcomes, and transmission dynamics of severe coronavirus disease 2019 in California and Washington: prospective cohort study. BMJ. 2020;369:m1923. PubMed https://doi.org/10.1136/bmj.m1923

34 Tindale L, Stockdale JE, Coombe M, Garlock E, Lau WYV, Saraswat M, et al. Evidence for transmission of COVID-19 prior to symptom onset. eLife. 2020;9:e57149. https://doi.org/10.7554/eLife.57149

35. Vaccines and Related Biological Products Advisory Committee. December 10, 2020 meeting briefing document—FDA. 2020 [cited 2021 May 11]. https://www.fda.gov/media/144245

36. Vaccines and Related Biological Products Advisory Committee December 17, 2020 meeting briefing document addendum—sponsor. 2020 [cited 2021 May 11]. https://www.fda.gov/media/144453

37. US Department of Defense. Operation Warp Speed leaders say 20 million COVID-19 vaccines may be available this month. 2020 [cited 2021 Jan 13].

https://www.defense.gov/Explore/News/Article/Article/2432663/operation-warp-speed-leaders-say-20-million-covid-19-vaccines-may-be-available/

38. Vaccines and Related Biological Products Advisory Committee December 17, 2020 meeting briefing document—FDA. 2020 [cited 2021 May 11]. https://www.fda.gov/media/144434

39. King AA, Nguyen D, Ionides EL. Statistical inference for partially observed Markov processes via the R package pomp. J Stat Softw. 2016;69:1–43.

40. Tec M, Lachmann M, Fox SJ, Pasco R, Woody SN, Starling J, et al. Austin COVID-19 transmission estimates and healthcare projections. [cited 2021 Feb 5]. https://sites.cns.utexas.edu/sites/default/files/cid/files/austin_dashboard_report_071520.pdf

41. Novavax COVID-19 vaccine demonstrates 89.3% efficacy in UK phase 3 trial. 2021 [cited 2021 Feb 10]. https://ir.novavax.com/news-releases/news-release-details/novavax-covid-19-vaccine-demonstrates-893-efficacy-uk-phase-3

42. Skowronski DM, De Serres G, Gruber WC. Safety and efficacy of the BNT162b2 mRNA COVID-19 vaccine. [Reply]. N Engl J Med. 2021;384:1576–8. PubMed https://doi.org/10.1056/NEJMc2036242

Appendix Table 1. Percent of deaths from coronavirus disease averted after January 15, 2021 by type of vaccination rollout for infection-blocking or symptom-blocking vaccines*

	January 15 rollout			February 15 rollout		
Vaccine schema	50% uptake	70% uptake	90% uptake	50% uptake	70% uptake	90% uptake
Infection-blocking 2 dose						
No priorities	48 (43–53)	48 (42–54)	48 (43–53)	30 (25–36)	30 (25–35)	31 (24 −36)
Adults >65 years of age	49 (44–54)	50 (45–55)	51 (45–55)	32 (24–37)	33 (27–38)	33 (27–39)
High-risk adults	52 (46–56)	54 (48–58)	54 (50–59)	34 (27–39)	35 (30–40)	36 (30–41)
Adults >65 years of age + high risk	51 (46–56)	52 (48–58)	53 (48–58)	33 (29–39)	34 (27–40)	36 (29–40)
10 Phase	52 (47–56)	54 (49–58)	56 (51–60)	34 (28–40)	36 (30–42)	38 (32–43)
Symptom-blocking 2 dose						
No priorities	32 (25–37)	32 (27–38)	33 (26–38)	20 (13–26)	19 (13–25)	19 (12–25)
Adults >65 years of age	36 (30–42)	40 (34–44)	42 (38–46)	23 (18–29)	26 (19–32)	28 (20–33)
High-risk adults	38 (32–43)	41 (35–47)	44 (39–49)	25 (18–30)	27 (21–33)	29 (24–34)
Adults >65 years of age + high risk	38 (32–43)	43 (38–49)	45 (41–50)	25 (19–30)	28 (22–33)	30 (24–35)
10 Phase	40 (35–45)	46 (40–51)	51 (45–56)	27 (21–33)	32 (25–37)	34 (29–39)
Infection-blocking 1 dose						
No priorities	65 (61–68)	65 (62–69)	65 (63–69)	44 (39–49)	44 (40–49)	44 (40–50)
Adults >65 years of age	66 (62–69)	66 (63–70)	68 (63–71)	45 (41–50)	47 (42–52)	47 (43–52)
High-risk adults	66 (62–69)	68 (64–72)	70 (66–73)	46 (41–52)	48 (43–53)	49 (43–54)
Adults >65 years of age + high risk	66 (63–70)	68 (64–71)	69 (65–73)	46 (41–51)	48 (43–53)	49 (44–54)
10 Phase	66 (63–70)	69 (66–73)	71 (67–74)	47 (41–51)	49 (44–54)	51 (46–55)
Symptom-blocking 1 dose						
No priorities	46 (39–50)	50 (45–55)	51 (47–56)	31 (25–36)	33 (27–39)	34 (28–38)
Adults >65 years of age	48 (42–52)	54 (49–58)	57 (52–61)	33 (28–38)	37 (32–43)	39 (33–45)
High-risk adults	49 (43–53)	55 (49–60)	58 (54–63)	34 (29–39)	38 (33–43)	41 (35–45)
Adults >65 years of age + high risk	49 (43–53)	55 (51–60)	60 (55–64)	34 (28–39)	39 (33–43)	42 (36–46)
10 Phase	50 (45–54)	57 (53–62)	63 (58–67)	35 (29–40)	41 (36–46)	45 (39–50)

*Values are medians and 95% confidence intervals based on 200 pairs of stochastic simulations. Deaths averted are computed by comparing a simulation of the specified vaccine strategy to a simulation without vaccination.

Appendix Table 2. Percent of deaths from coronavirus disease averted after January 15, 2021 by type of vaccination rollout for hybrid scenarios*

Vaccine schema	January 15 rollout			February 15 rollout		
	50% uptake	70% uptake	90% uptake	50% uptake	70% uptake	90% uptake
Hybrid 2 dose						
No priorities	47 (41–51)	46 (41–51)	47 (41–52)	30 (22–35)	29 (23–35)	29 (22–35)
Adults >65 years of age	47 (43–53)	48 (43–54)	50 (43–55)	31 (25–36)	32 (25–37)	33 (27–38)
High-risk adults	50(43–55)	52(46–57)	53(47–58)	33 (27–38)	34(27–39)	35(29–40)
Adults >65 years of age + high risk	50 (45–54)	51 (46–56)	53 (46–57)	32 (25–37)	34 (27–39)	34 (28–40)
10 Phase	50 (45–55)	53 (48–58)	56 (51–60)	34 (28–39)	36 (30–41)	37 (31–44)
Hybrid 1 dose						
No priorities	63 (59–67)	64 (60–68)	64 (59–67)	43 (37–47)	43 (37–48)	44 (38–48)
Adults >65 years of age	64 (59–68)	65 (61–69)	66 (62–70)	44 (39–49)	45 (40–50)	46 (41–52)
High-risk adults	65 (61–68)	67 (62–70)	68 (65–72)	45 (39–49)	47 (41–52)	48 (43–52)
Adults >65 years of age + high risk	64 (60–68)	67 (63–70)	68 (64–71)	45 (40–50)	47 (42–51)	48 (44–52)
10 Phase	65 (61–69)	68 (64–72)	70 (66–73)	46 (40–50)	48 (44–53)	50 (46–54)

*Values are medians and 95% confidence intervals based on 200 pairs of stochastic simulations. Deaths averted are computed by comparing a simulation of the specified vaccine strategy to a simulation without vaccination.

Appendix Table 3. Initial conditions, school calendar, and contact rates used in modeling of effects of vaccination by prioritization on mortality

Variable	Settings
Initial day of simulation	11/8/2020
Initial number infected	Based on estimates for Austin, Texas, given Appendix Table 11 (*5*)
Age-specific and day-specific contact rates*	Home, work, other and school matrices provided in Appendix Tables 7–10<br>Typical weekday = home + work + other + school<br>Weekends and holiday weekdays = home + other<br>Weekdays during non-holiday school breaks = home + work + other
School calendar	Austin Independent School District calendar (2019–2020, 2020–2021) (*6*)

*We assume the age-specific contact rates given in (*8*), which takes the contact numbers estimated through diary-based POLYMOD study in Europe (*9*) and extrapolates to the United States. The values in Appendix Tables 7–10 are the assumed daily contacts between each pair of age groups at home, school, work, and all other places, respectively. These contact matrices are used to adjust the transmission rate between age groups. The accuracy of the contact matrices is limited by (i) possible biases with the original diary-based study (*9*), (ii) assumptions made when projecting the original study to the U.S (*7*), and (iii) impacts of COVID-19 policies and perceptions on daily contact patterns.

Appendix Table 4. Epidemiologic parameters for modeling of effects of vaccination by prioritization on mortality*

Parameters	Best-guess values	Source
R_e: effective reproduction number	1.2	(*5*)
β: baseline transmission rate	0.0183	Derived by next-generation matrix method to yield R_e = 1.2 without prior immunity (*9*)
γ^A: recovery rate on asymptomatic compartment†	0.1587	(*10*)
γ^Y: recovery rate on symptomatic non-treated compartment	0.25	(*10*)
τ: symptomatic proportion (%)	57	(*11*)
σ: exposed rate	0.3448	(*12*)
P: proportion of infections occurring in pre-symptomatic period (%)	44	(*10*)
ω^P: relative infectiousness of infectious individuals in compartment I^P	1.3669	$\omega^P = (\frac{YHR}{\eta} + \frac{1-YHR}{\gamma^Y})\frac{\omega^Y \rho P}{1-P}$
ω^A: relative infectiousness of infectious individuals in compartment I^A	0.67	(*10*)
ρ: symptom onset rate	0.43478	2.3 d average pre-symptomatic period (*10*)
IFR: age-stratified infection fatality ratio (%)	Overall: [0.0016, 0.00495, 0.08428, 1.00011, 3.37149]<br>Low risk: [0.00137, 0.00386, 0.06334, 0.60254, 1.73687]<br>High risk: [0.00412, 0.01157, 0.19001, 1.80762, 5.2106]	Age adjusted from Verity et al., unpub. data, https://doi.org/10.1101/2020.03.09.20033357
YFR: age-stratified symptomatic fatality ratio (%)	Overall: [0.00281, 0.00868, 0.14785, 1.75458, 5.9149]<br>Low risk: [0.00241, 0.00677, 0.11112, 1.05709, 3.04713]<br>High risk: [0.00722, 0.0203, 0.33336, 3.17127, 9.1414]	$YFR = \frac{IFR}{\tau}$

Parameters	Best-guess values	Source
h: high-risk proportion, age specific (%)	[8.2825, 14.1121, 16.5298, 32.9912, 47.0568]	Based on CDC's list of high risk conditions for severe influenza; see Section 4 below (*13*–*29*)
rr: relative risk for hospitalization for high-risk versus low-risk persons in a given age group	3	Suggested by CDC for COVID-19 scenario projections (*30*) and consistent with COVID-19 risk factor studies (*31*)

*Values given as five-element vectors are age-stratified with values corresponding to 0–4, 5–17, 18–49, 50–64, 65+ year age groups
†We assume that the duration of the infectious period is the same for asymptomatic and symptomatic cases.

Appendix Table 5. Hospitalization parameters

Parameters	Value	Source
γ^H: recovery rate in hospitalized compartment	0.0935	Austin admissions and discharge data (Avg = 10.96. 95% CI = 9.37 to 12.76) (*32*,*33*)
YHR: symptomatic case hospitalization rate (%)	Overall: [0.07018, 0.07018, 4.73526, 16.32983, 25.54183]<br>Low risk: [0.0602, 0.05473, 3.55875, 9.83829, 13.15819]<br>High risk: [0.18061, 0.16419, 10.67625, 29.51487, 39.47457]	Age adjusted from Verity et al., unpub. data (https://doi.org/10.1101/2020.03.09.20033357)
π: rate of symptomatic individuals go to hospital, age-specific	Low risk: [0.0009, 0.0008, 0.0516, 0.1386, 0.1827]<br>High risk: [0.0027, 0.0024, 0.1499, 0.3818, 0.4903]	$\pi = \frac{\gamma^Y \cdot YHR}{\eta + (\gamma^Y - \eta)YHR}$
η: rate from symptom onset to hospitalized	0.1695	5.9 d average from symptom onset to hospital admission Tindale et al. (*34*)
μ: rate from hospitalized to death	0.1235	Austin admissions and discharge data (Avg = 7.8, 95% CI = 5.21 to 10.09) (*332*,*33*)
HFR: hospitalized fatality ratio, age specific (%)	[4, 12.365, 3.122, 10.745, 23.158]	$HFR = \frac{IFR}{YHR\tau}$
ν: death rate on hospitalized individuals, age specific	0.0617	$\nu = \frac{\gamma^H HFR}{\mu + (\gamma^H - \mu)HFR}$

Appendix Table 6. Vaccine parameters

Parameters	Value	Source
ve^f: efficacy of full course vaccination, infection-blocking or symptom blocking	After 1st and before 2nd dose:<br>$ve_1^f \sim Triangular(0.295, 0.524, 0.684)$<br>After 2nd dose:<br>$ve_2^f \sim Triangular(0.898, 0.948, 0.976)$	(*35*)
ve^s: vaccine efficacy - single dose only, infection-blocking or symptom blocking	After single dose only:<br>Main scenario<br>• $ve^s \sim Triangular(0.756, 0.82, 0.869)$<br>Sensitivity analysis<br>• $ve^s \sim Triangular(0.295, 0.524, 0.684)$<br>• $ve^s \sim Triangular(0.465, 0.685, 0.815)$<br>• $ve^s \sim Triangular(0.69, 0.926, 0.983)$	(*35*)
$ve^{h,f}$: efficacy of full course vaccination, hybrid model	Efficacy against infection<br>• after 1st and before 2nd dose, $ve_{1,i}^{h,f}$ = 0.524<br>• after 2nd dose, $ve_{2,i}^{h,f}$ =0.67<br>Efficacy against symptomatic diseases<br>• after 1st and before 2nd dose, $ve_{1,y}^{h,f}$ = 0<br>• after 2nd dose,<br>$ve_{2,y}^{h,f} \sim Triangular(0.691, 0.82, 0.927)$	(*35*,*36*)
$ve^{h,s}$: vaccine efficacy - single dose only, hybrid model	Efficacy against infection: $ve_i^{h,s}$ = 0.67<br>Efficacy against symptomatic infection:<br>$ve_y^{h,s} \sim Triangular(0.261, 0.455, 0.603)$	(*35*,*36*)
vu: vaccine uptake rate	0.5, 0.7, 0.9	0.5 is based on a survey conducted by PEW Research Center in September (*2*); 0.7 is based on a survey conducted by PEW Research Center in May (*2*); 0.9 is hypothetical
α^{ii}: second dose return rate	0.8	Assumption

Parameters	Value	Source
Vaccine rollout schedule	10M nationwide per week and adjusted by Austin MSA population	(*37*) and discussion with CDC
δ: delay between 1st and 2nd dose	$1/\delta$ = 28 d	(*38*)
δ^{i}, δ^{ii}: rate of acquiring immunity after the first and second dose	$1/\delta^{i} = 1/\ \delta^{ii} = 14$ d	(*38*)

Appendix Table 7. Home contact matrix (daily number contacts by age group at home)

Age group	0–4 y	5–17 y	18–49 y	50–64 y	≥65 y
0–4 y	0.5	0.9	2.0	0.1	0.0
5–17 y	0.2	1.7	1.9	0.2	0.0
18–49 y	0.2	0.9	1.7	0.2	0.0
50–64 y	0.2	0.7	1.2	1.0	0.1
≥65 y	0.1	0.7	1.0	0.3	0.6

Appendix Table 8. School contact matrix (daily number contacts by age group at school)

Age group	0–4 y	5–17 y	18–49 y	50–64 y	≥65 y
0–4 y	1.0	0.5	0.4	0.1	0.0
5–17 y	0.2	3.7	0.9	0.1	0.0
18–49 y	0.0	0.7	0.8	0.0	0.0
50–64 y	0.1	0.8	0.5	0.1	0.0
≥65 y	0.0	0.0	0.1	0.0	0.0

Appendix Table 9. Work contact matrix (daily number contacts by age group at work)

Age group	0–4 y	5–17 y	18–49 y	50–64 y	≥65 y
0–4 y	0.0	0.0	0.0	0.0	0.0
5–17 y	0.0	0.1	0.4	0.0	0.0
18–49 y	0.0	0.2	4.5	0.8	0.0
50–64 y	0.0	0.1	2.8	0.9	0.0
≥65 y	0.0	0.0	0.1	0.0	0.0

Appendix Table 10. Others contact matrix (daily number contacts by age group at other locations)

Age group	0–4 y	5–17 y	18–49 y	50–64 y	≥65 y
0–4 y	0.7	0.7	1.8	0.6	0.3
5–17 y	0.2	2.6	2.1	0.4	0.2
18–49 y	0.1	0.7	3.3	0.6	0.2
50–64 y	0.1	0.3	2.2	1.1	0.4
≥65 y	0.0	0.2	1.3	0.8	0.6

Appendix Table 11. Initial states of model compartments*

Age	Risk	S	E	I^A	I^P	I^Y	I^H	R	D
0–4 y	High	8,809	7	5	3	5	0	524	0
	Low	121,035	95	87	42	73	0	7,190	0
5–17 y	High	33,884	40	39	21	33	0	3,418	0
	Low	296,425	372	343	169	279	0	29,580	1
18–49 y	High	142,443	165	143	70	140	35	13,158	26
	Low	834,952	949	884	442	746	21	77,836	16
50–64 y	High	100,333	102	84	44	87	42	7,262	255
	Low	231,158	219	201	97	171	9	17,370	57
≥65 y	High	100,101	45	40	21	43	20	3,261	236
	Low	127,788	60	55	26	51	2	4,485	31

*Values indicate the number of individuals in each age-risk group compartment at the start of the simulations.

Appendix Table 12. High-risk conditions for influenza and data sources for prevalence estimation

Condition	Data source
Cancer (except skin), chronic kidney disease, COPD, coronary heart disease, stroke, asthma, diabetes	CDC 500 Cities (*16*)
HIV/AIDS	CDC HIV Surveillance report (*17*)
Obesity	CDC 500 Cities (*16*), Sturm and Hattori (*20*), Morgan et al. (*21*)
Pregnancy	National Vital Statistics Reports (*27*) and abortion data (*28*)

Appendix Table 13. Comparison between published national estimates and Austin-Round Rock MSA estimates of the percent of the population at high risk for influenza/COVID-19 complications*

Age group	National estimates (*25*)	Austin	Pregnant women (proportion of age group)
0–6 mo	NA	6.8	–
6 mo–4 y	6.8	7.4	–
5–9 y	11.7	11.6	–
10–14 y	11.7	13.0	–
15–19 y	11.8	13.3	1.7
20–24 y	12.4	10.3	5.1
25–34 y	15.7	13.5	7.8
35–39 y	15.7	17.0	5.1
40–44 y	15.7	17.4	1.2
45–49 y	15.7	17.7	–
50–54 y	30.6	29.6	–
55–60 y	30.6	29.5	–
60–64 y	30.6	29.3	–
65–69 y	47.0	42.2	–
70–74 y	47.0	42.2	–
≥75 y	47.0	42.2	–

*Proportions for Austin exclude pregnancy. NA, not available.

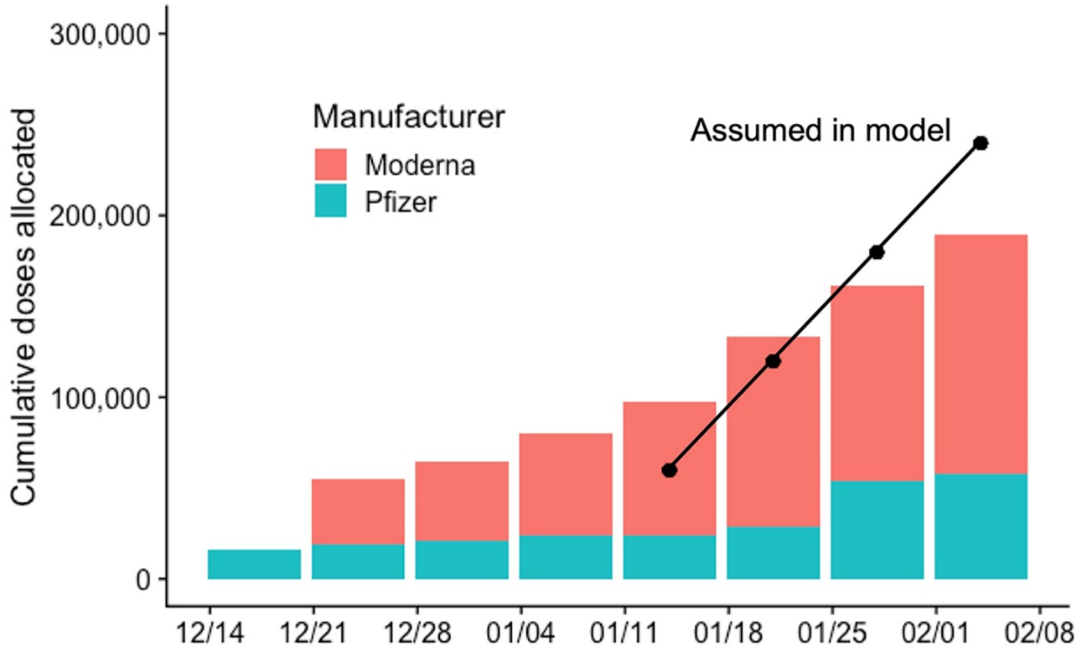

Appendix Figure 1. COVID-19 vaccine doses allocated to the Austin-Round Rock Metropolitan Statistical Area in comparison to the model assumption (*1*). Red represents doses manufactured by Moderna and green represents vaccines manufactured by Pfizer-BioNTech. Black points indicate the rollout assumed by the model.

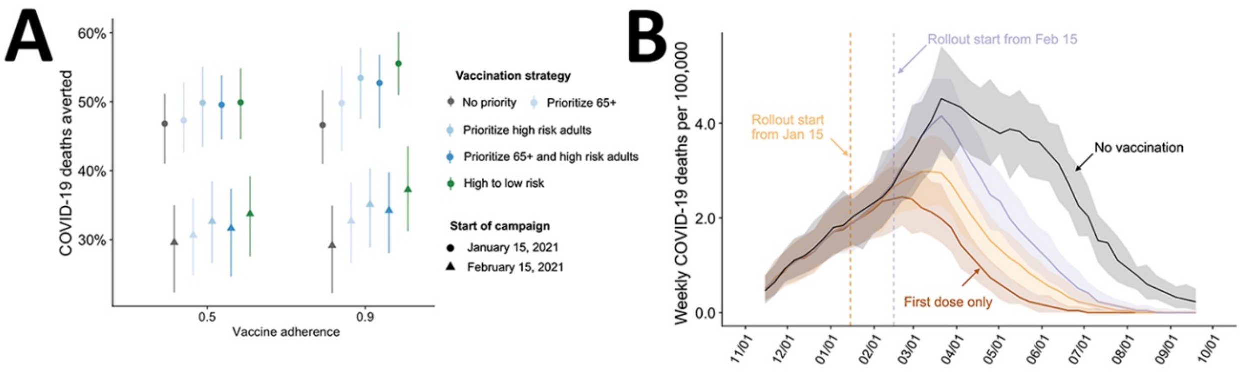

Appendix Figure 2. Projected COVID-19 mortality in the Austin-Round Rock Metropolitan Statistical Area for November 8, 2020–September 17, 2021 under various vaccine rollout scenarios. A) COVID-19 deaths averted after January 15, 2021 for a hybrid type of vaccination protection under combinations of: vaccine uptake, either 50% or 90% (x-axis); rollout dates, either January 15 (circles) or February 15 (triangles); and risk prioritization, either no priority (gray), prioritize all adults over 65 y (light blue), adults with high-risk comorbidities (medium blue), or the combination of the two (dark blue), or a 10-phase risk-ordered strategy (green) that sequentially vaccinates >65 y high risk, 50–64 y high risk, >65 y low risk, 18–49 y high risk, 50–64 y low risk, 18–49 y low risk, 0–4 y high risk, 5–17 y high risk, 0–4 y low risk, 5–17 y low risk. Points and whiskers indicate the median and 95% CI across 200 paired stochastic simulations. B) Weekly incident COVID-19 deaths per 100,000 assuming intermediate (70%) uptake (*2*) without vaccine (black) or under a 10-phase risk-based rollout of a 95% efficacious infection-blocking vaccine, starting either January 15 (orange) or February 15 (purple). The brown line assumes that only first doses are administered starting January 15. Solid lines and shading indicate the median and 95% CI across 200 stochastic simulations.

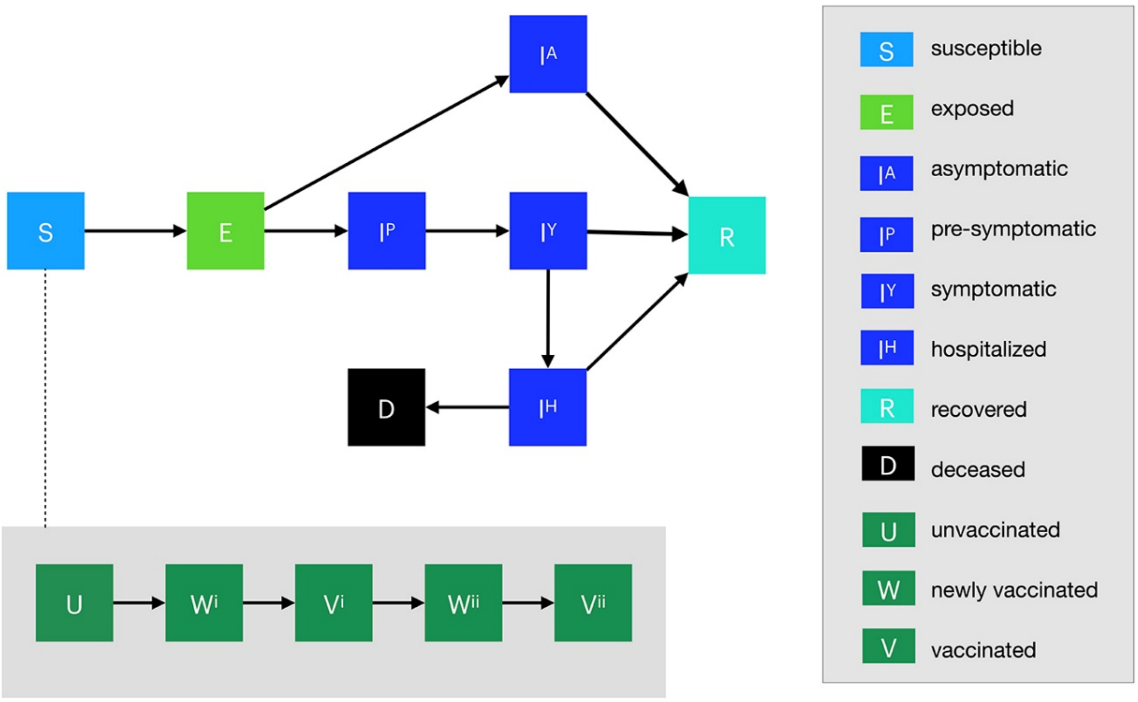

Appendix Figure 3. Compartmental model of COVID-19 transmission in the Austin-Round Rock Metropolitan Statistical Area for November 8, 2020–September 17, 2021 under various vaccine rollout scenarios. Each age-risk subgroup is modeled with a separate set of compartments. Upon infection, susceptible individuals (S) progress to exposed (E) and then to either pre-symptomatic infectious (I^P) or asymptomatic infectious (I^A). All asymptomatic cases eventually progress to a recovered class where they remain protected from future infection (R); pre-symptomatic cases progress to symptomatic (I^Y) then are either hospitalized (I^H) or recover. Mortality (D) varies by age group and risk group and is assumed to be preceded by hospitalization. Within each compartment, individuals are divided by vaccination status: unvaccinated (U), newly vaccinated with the first dose (W^i), vaccinated with the first dose (V^i), newly vaccinated with the second dose (W^{ii}), and fully vaccinated with the second dose (V^{ii}). We model stochastic transitions between compartments and sample vaccine efficacy parameters from distributions to capture uncertainty, while keeping all other parameters fixed.

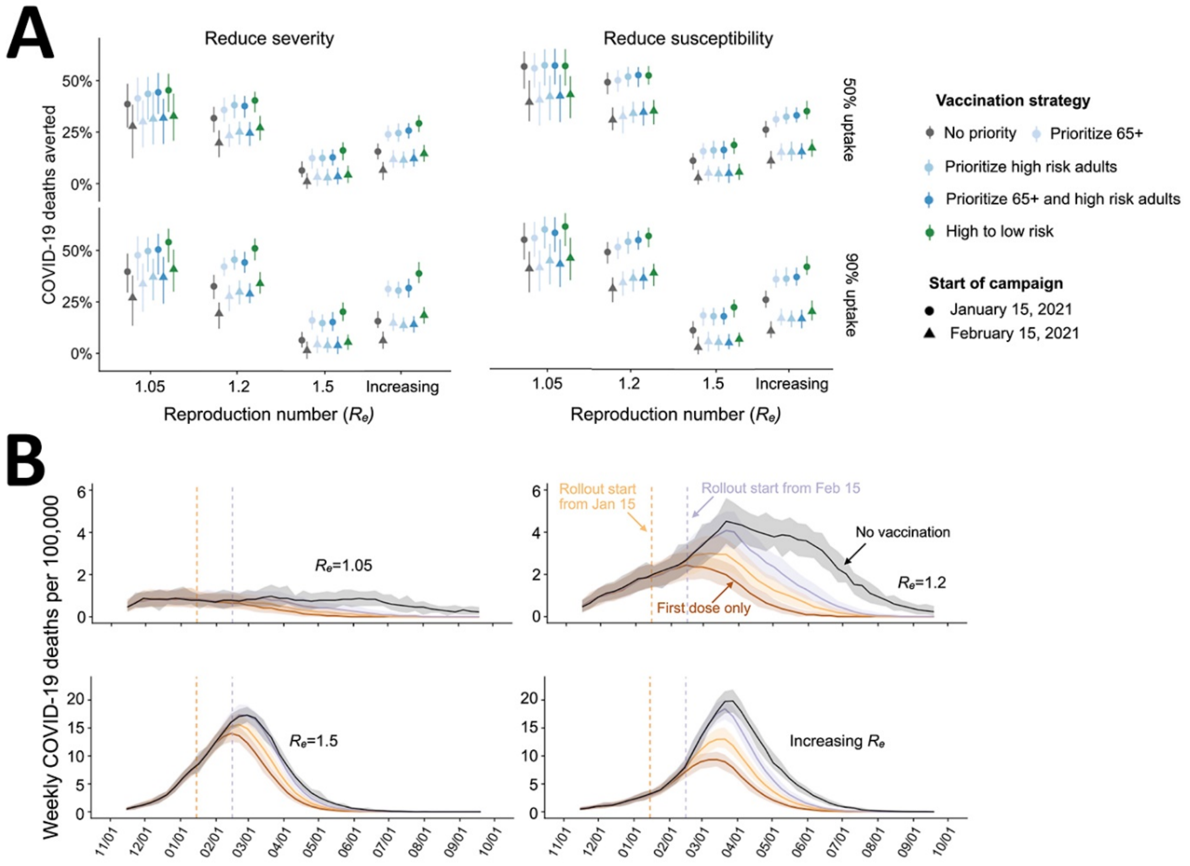

Appendix Figure 4. 1. Projected COVID-19 mortality in the Austin-Round Rock Metropolitan Statistical Area for November 8, 2020–September 17, 2021 under different vaccination scenarios, assuming either R_e = 1.05, R_e = 1.2, R_e = 1.5, or R_e increases linearly from 1.2 to 1.8 by May 1, 2021, and remains at 1.8 thereafter. A) COVID-19 deaths averted after January 15, 2021 under combinations of: vaccine uptake, either 50% (top) or 90% (bottom); type of protection, either symptom blocking (left) or infection blocking (right); rollout dates, either January 15 (circles) or February 15 (triangles); and risk prioritization, either no priority (gray), prioritize all adults over 65 y (light blue), adults with high-risk comorbidities (medium blue), or the combination of the two (dark blue), or a 10-phase risk-ordered strategy (green) that sequentially vaccinates >65 y high risk, 50–64 y high risk, >65 y low risk, 18–49 y high risk, 50–64 y low risk, 18–49 y low risk, 0–4 y high risk, 5–17 y high risk, 0–4 y low risk, 5–17 y low risk. Points and whiskers indicate the median and 95% CI across 200 paired stochastic simulations. B) Weekly incident COVID-19 deaths per 100,000 population assuming intermediate (70%) uptake (*2*) without vaccine (black) or under a 10-phase risk-based rollout of a 95% efficacious infection-blocking vaccine, starting either January 15 (orange) or February 15 (purple), for the indicated R_e scenario. The brown lines assume that only first doses are administered starting January 15. Solid lines and shading indicate the median and 95% CI across 200 stochastic simulations.

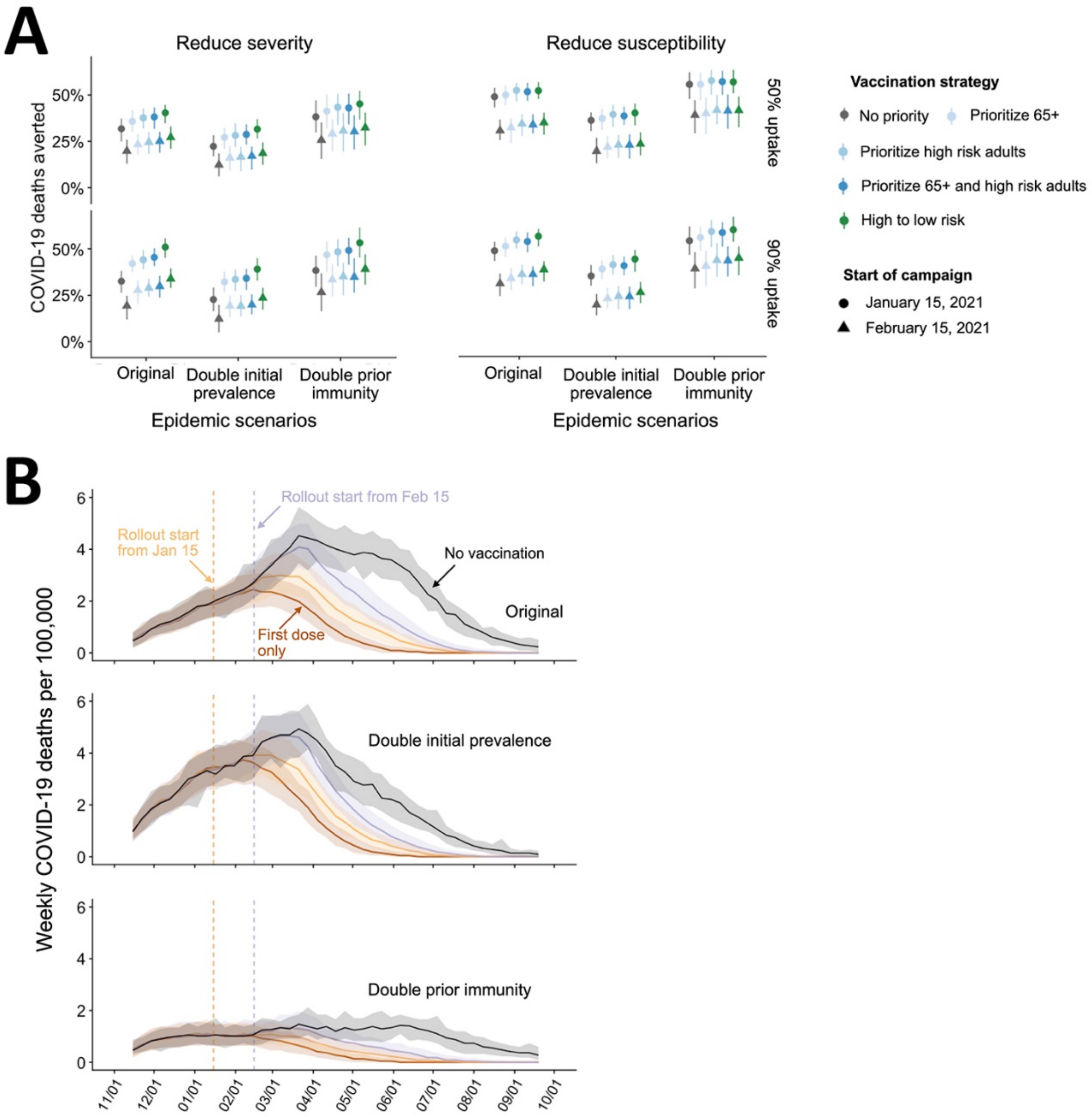

Appendix Figure 5. Projected COVID-19 mortality in the Austin-Round Rock Metropolitan Statistical Area for November 8, 2020–September 17, 2021 under different vaccine rollout scenarios, assuming a higher initial prevalence (0.6%) or prior immunity (15.2%). A) COVID-19 deaths averted after January 15, 2021 under combinations of: vaccine uptake, either 50% (top) or 90% (bottom); type of protection, either symptom blocking (left) or infection blocking (right); initial conditions (x-axis); rollout dates, either January 15 (circles) or February 15 (triangles); and risk prioritization (colors). Points and whiskers indicate the median and 95% CI across 200 paired stochastic simulations. B) Weekly incident COVID-19 deaths per 100,000 assuming intermediate (70%) uptake (*2*) without vaccine (black) or under a 10-phase risk-based rollout of a 95% efficacious infection-blocking, starting either January 15 (orange) or February 15 (purple), under the original scenario (top), double initial prevalence (middle), and double prior immunity (bottom). The brown line assumes that only first doses are administered starting January 15. Solid lines and shading indicate the median and 95% CI across 200 stochastic simulations.

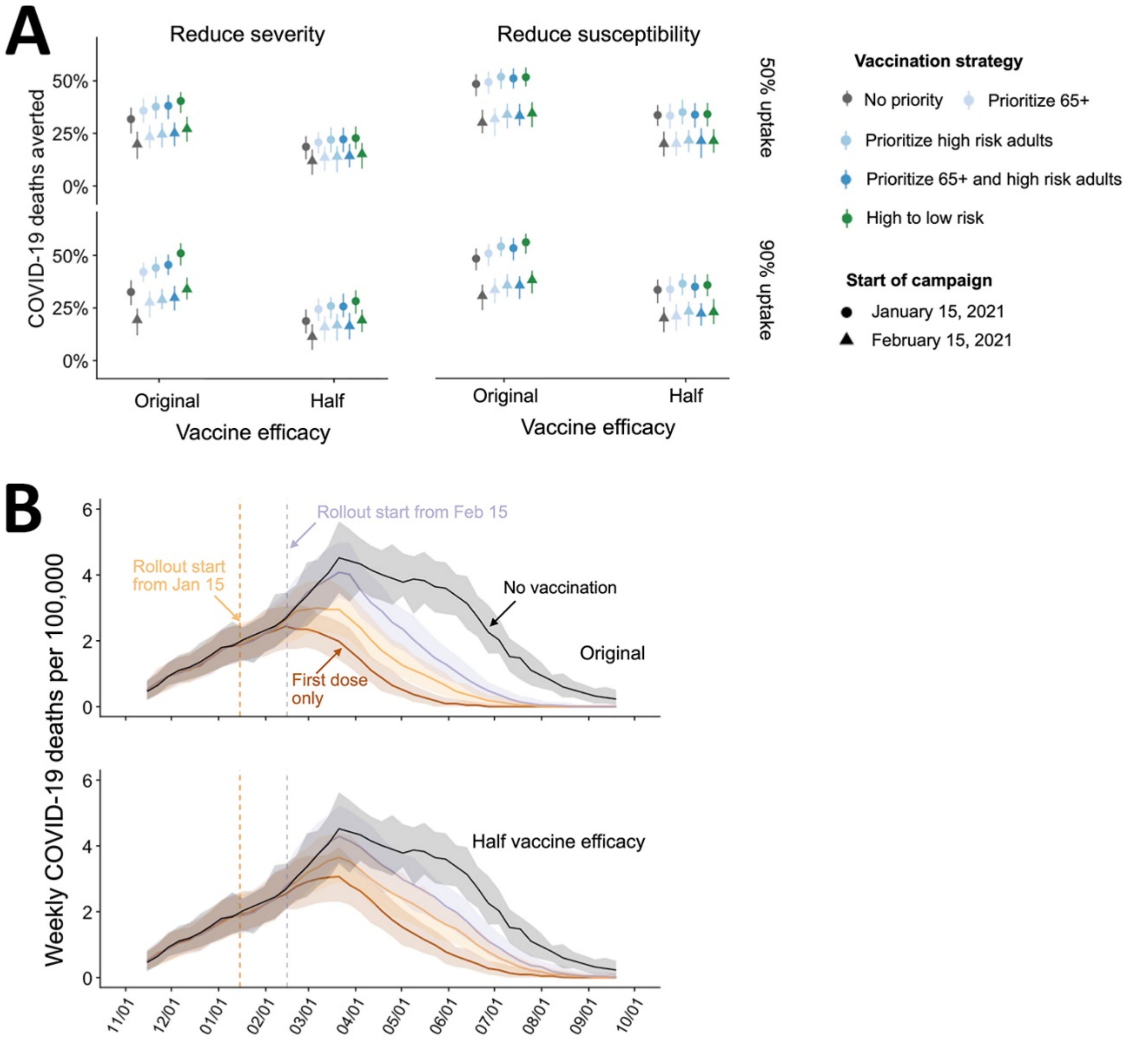

Appendix Figure 6. Projected COVID-19 in the Austin-Round Rock Metropolitan Statistical Area for November 8, 2020–September 17, 2021 under different vaccine rollout scenarios, assuming a 50% reduction in vaccine efficacy. A) COVID-19 deaths averted after January 15, 2021 under combinations of: vaccine uptake, either 50% (top) or 90% (bottom); type of protection, either symptom blocking (left) or infection blocking (right); vaccine efficacy, either original or half of original (x-axis); rollout dates, either January 15 (circles) or February 15 (triangles); and risk prioritization (colors). Points and whiskers indicate the median and 95% CI across 200 paired stochastic simulations. B) Weekly incident COVID-19 deaths per 100,000 assuming intermediate (70%) uptake (*2*) without vaccine (black) or under a 10-phase risk-based rollout of a 95% efficacious infection-blocking, starting either January 15 (orange) or February 15 (purple) with original vaccine efficacy (top) or 50% lower vaccine efficacy (bottom). The brown line assumes that only first doses are administered starting January 15. Solid lines and shading indicate the median and 95% CI across 200 stochastic simulations.

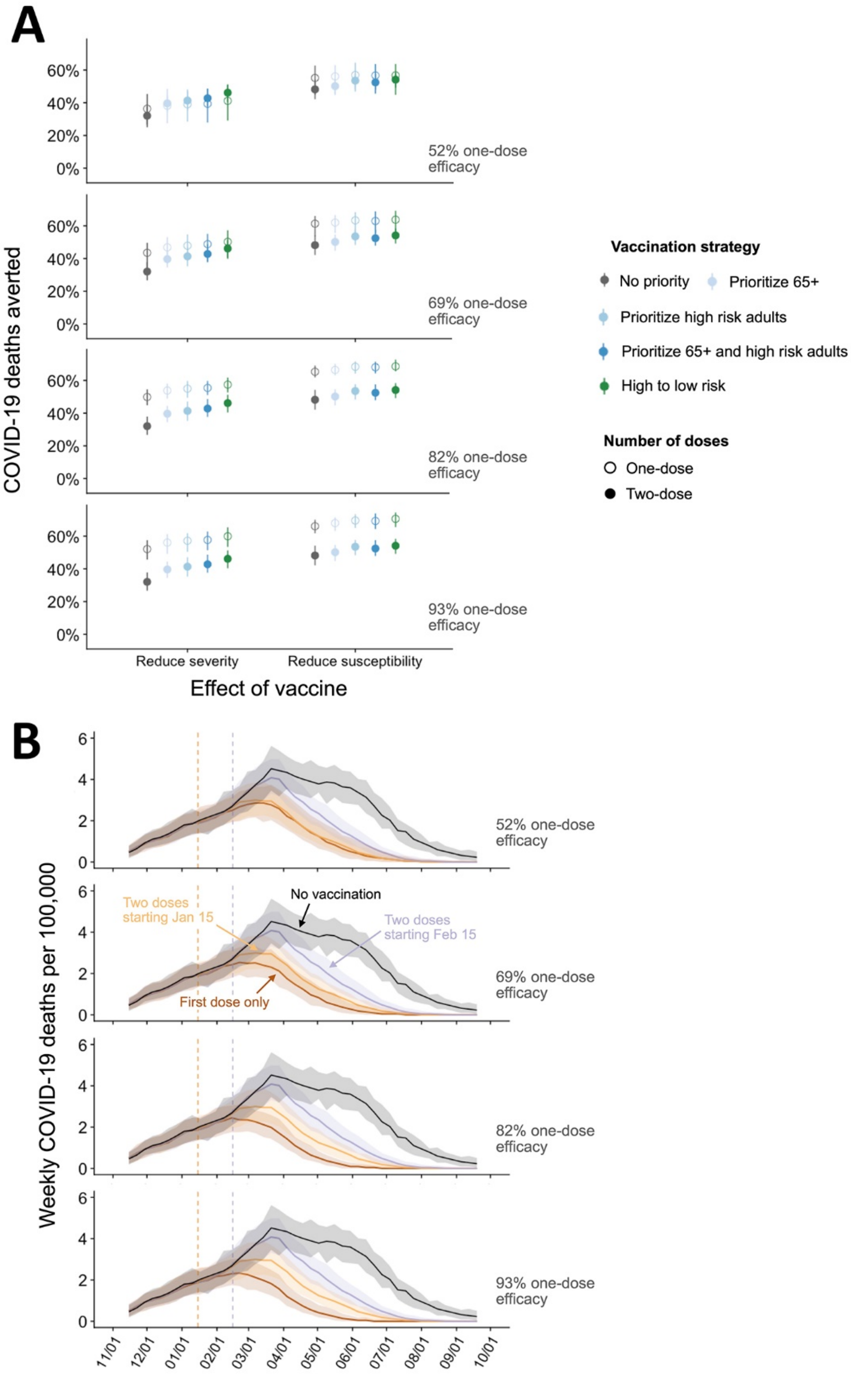

Appendix Figure 7. Projected COVID-19 mortality in the Austin-Round Rock Metropolitan Statistical Area for November 8, 2020–September 17, 2021 under different assumptions regarding the efficacy of a single dose. In both panels, the graphs from top to bottom assume single-dose efficacies of 52%, 69%, 82%

(original) and 93%, based on ref (*42*). A) COVID-19 deaths averted after January 15, 2021 under combinations of: type of protection, either symptom blocking (reducing severity) or infection blocking (reducing susceptibility); 1-dose (hollow) or 2-dose (solid); and risk prioritization (colors). All projections assume intermediate (70%) vaccine uptake (*2*) starting January 15. Points and whiskers indicate the median and 95% CI across 200 paired stochastic simulations. B) Weekly incident COVID-19 deaths per 100,000 population assuming intermediate (70%) uptake (*2*) without vaccine (black) or under a ten-phase risk-based rollout of a 95% efficacious infection-blocking, starting either January 15 (orange) or February 15 (purple). The brown line assumes that only first doses are administered starting January 15. Solid lines and shading indicate the median and 95% CI across 200 stochastic simulations.

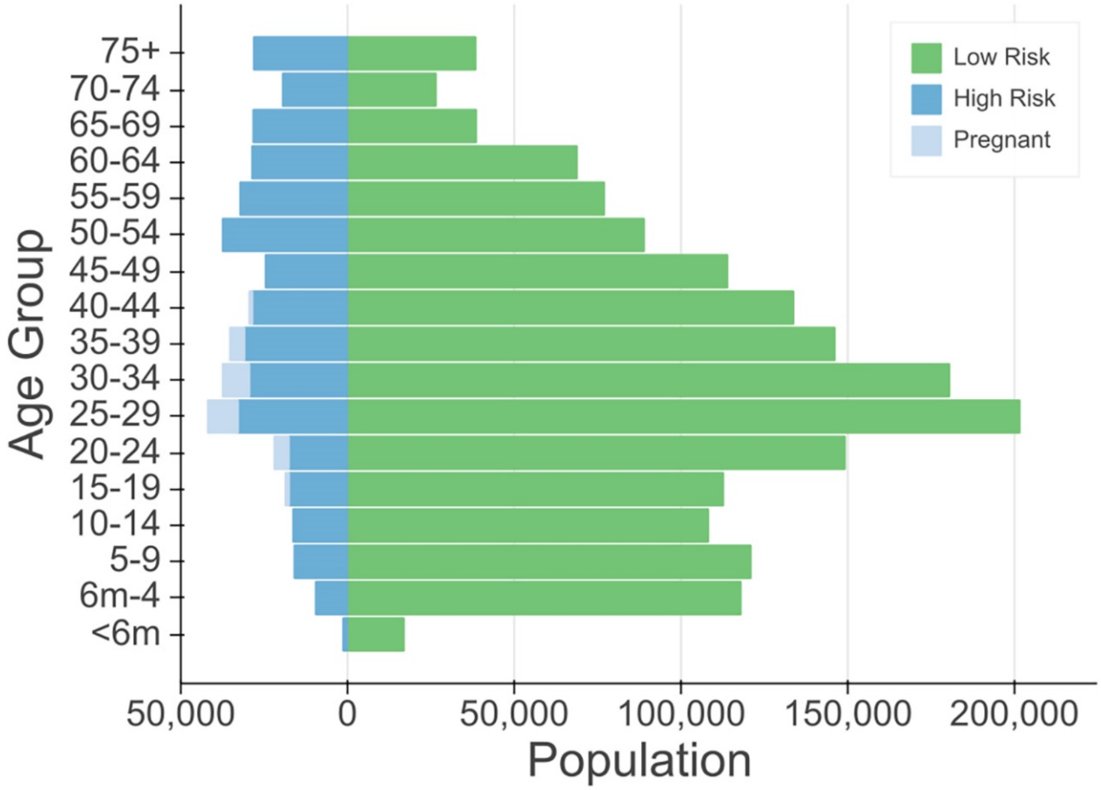

Appendix Figure 8. Demographic and risk composition of the Austin-Round Rock Metropolitan Statistical Area. Bars indicate age-specific population sizes, separated by low risk, high risk, and pregnant. High risk is defined as persons with cancer, chronic kidney disease, COPD, heart disease, stroke, asthma, diabetes, HIV/AIDS, and morbid obesity, as estimated from the CDC 500 Cities Project (*16*), reported HIV prevalence (*19*), and reported morbid obesity prevalence (*20,21*), corrected for multiple conditions. The population of pregnant women is derived using the CDC's method combining fertility, abortion and fetal loss rates (*27–29*).
